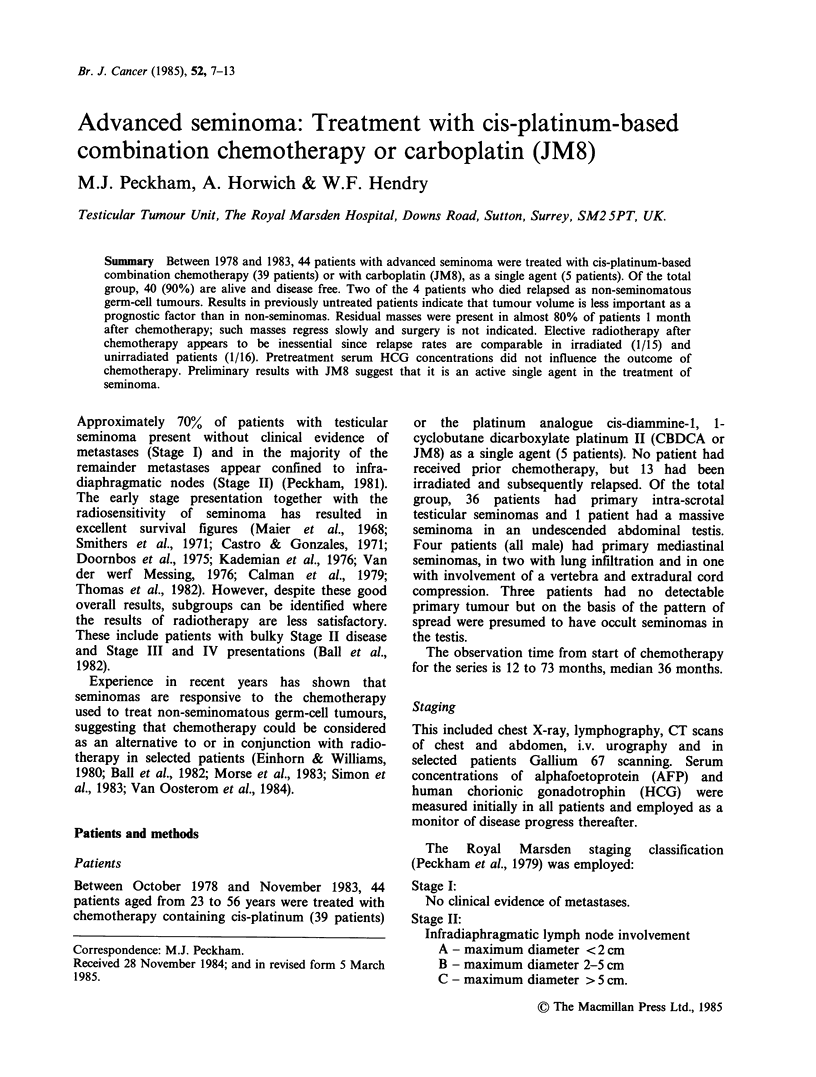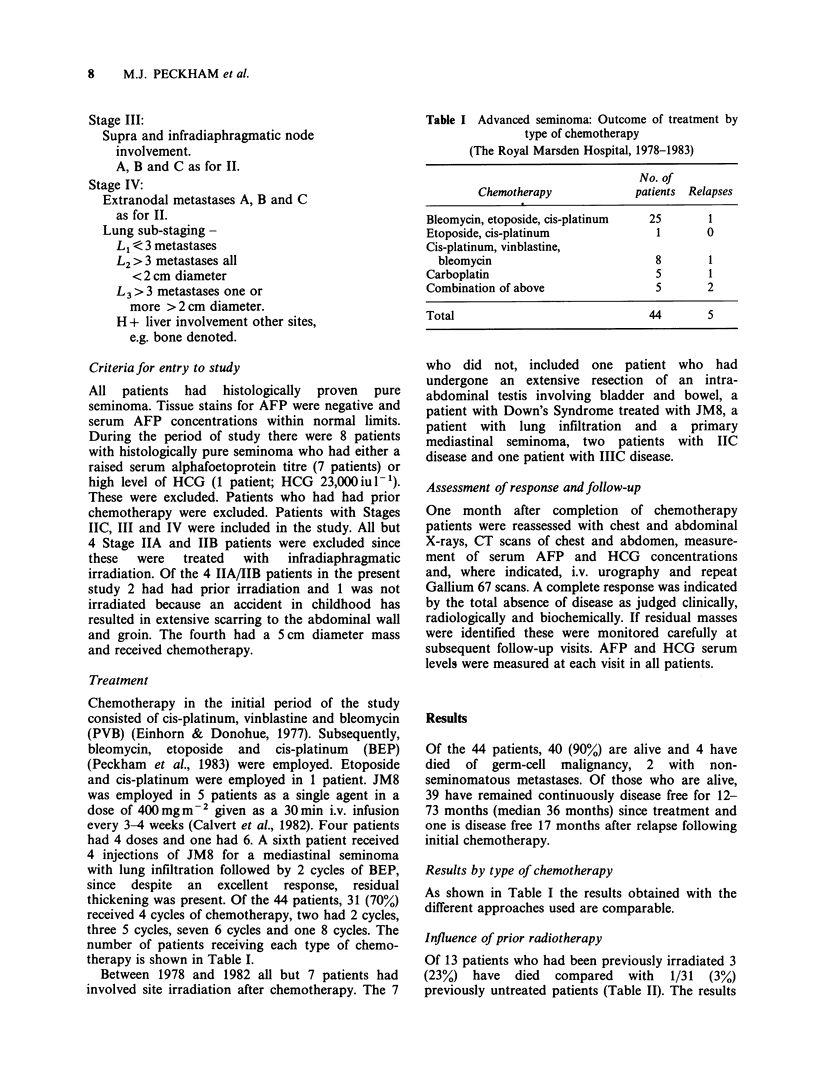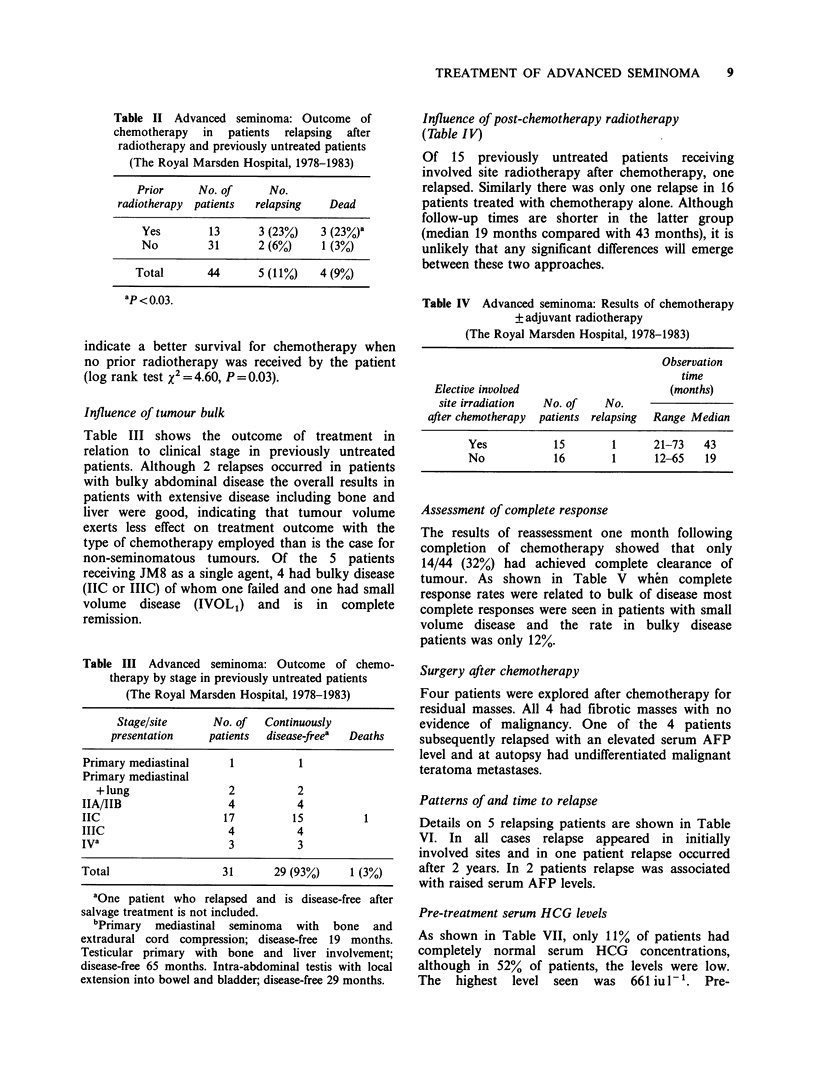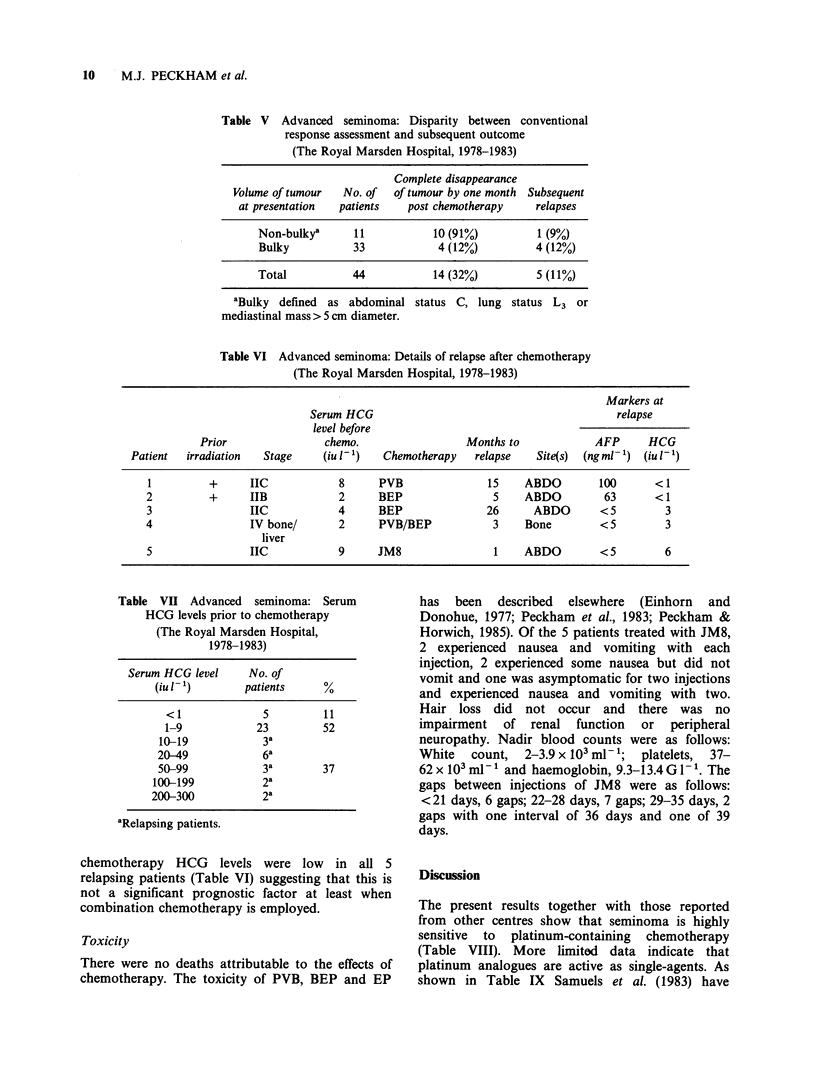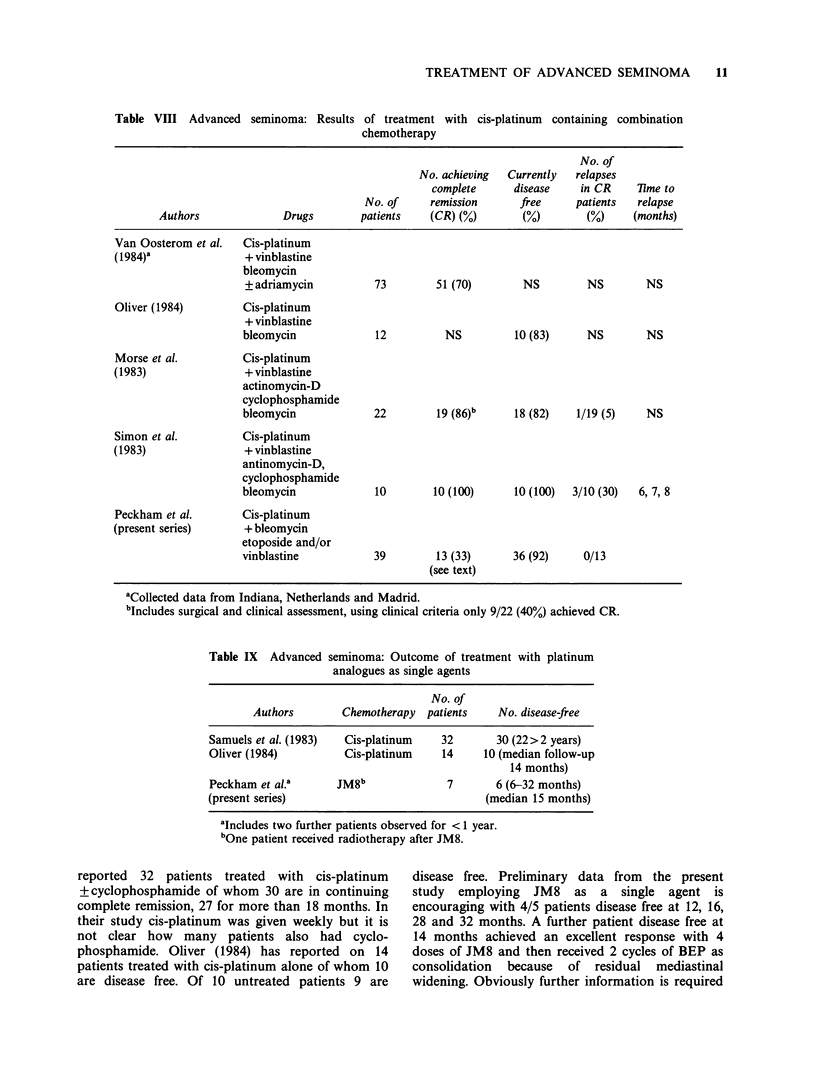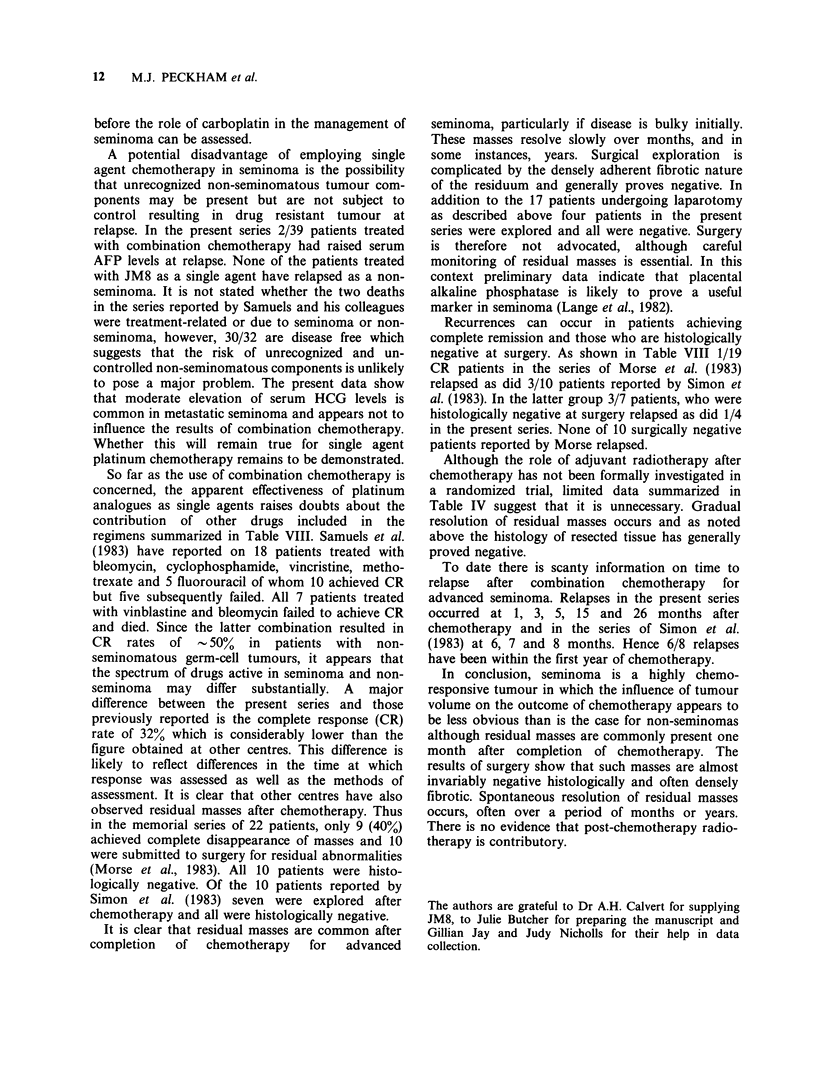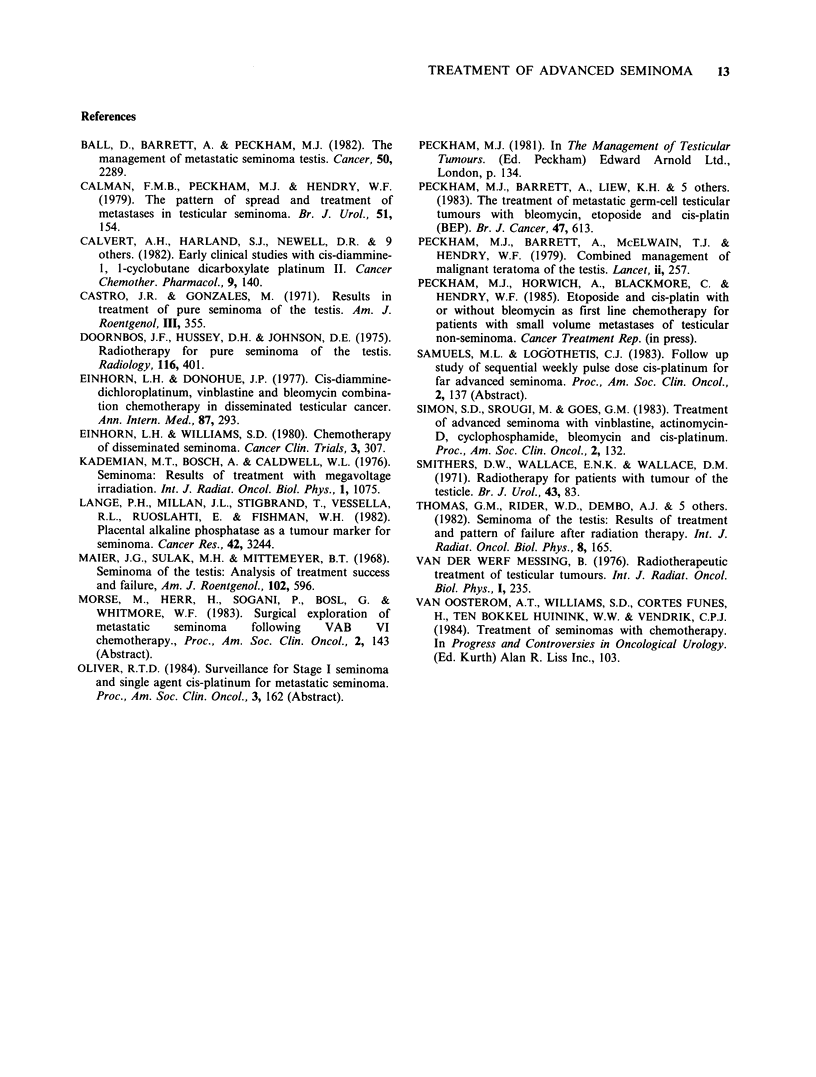# Advanced seminoma: treatment with cis-platinum-based combination chemotherapy or carboplatin (JM8).

**DOI:** 10.1038/bjc.1985.141

**Published:** 1985-07

**Authors:** M. J. Peckham, A. Horwich, W. F. Hendry

## Abstract

Between 1978 and 1983, 44 patients with advanced seminoma were treated with cis-platinum-based combination chemotherapy (39 patients) or with carboplatin (JM8), as a single agent (5 patients). Of the total group, 40 (90%) are alive and disease free. Two of the 4 patients who died relapsed as non-seminomatous germ-cell tumours. Results in previously untreated patients indicate that tumour volume is less important as a prognostic factor than in non-seminomas. Residual masses were present in almost 80% of patients 1 month after chemotherapy; such masses regress slowly and surgery is not indicated. Elective radiotherapy after chemotherapy appears to be inessential since relapse rates are comparable in irradiated (1/15) and unirradiated patients (1/16). Pretreatment serum HCG concentrations did not influence the outcome of chemotherapy. Preliminary results with JM8 suggest that it is an active single agent in the treatment of seminoma.


*Br. J. Cancer* (1985), **52**, 7–13

### Advanced seminoma: Treatment with cis-platinum-based combination chemotherapy or carboplatin (JM8)

M.J. Peckham, A. Horwich & W.F. Hendry

*Testicular Tumour Unit, The Royal Marsden Hospital, Downs Road, Sutton, Surrey, SM2 5PT, UK.*

**Summary** Between 1978 and 1983, 44 patients with advanced seminoma were treated with cis-platinum-based combination chemotherapy (39 patients) or with carboplatin (JM8), as a single agent (5 patients). Of the total group, 40 (90%) are alive and disease free. Two of the 4 patients who died relapsed as non-seminomatous germ-cell tumours. Results in previously untreated patients indicate that tumour volume is less important as a prognostic factor than in non-seminomas. Residual masses were present in almost 80% of patients 1 month after chemotherapy; such masses regress slowly and surgery is not indicated. Elective radiotherapy after chemotherapy appears to be inessential since relapse rates are comparable in irradiated (1/15) and unirradiated patients (1/16). Pretreatment serum HCG concentrations did not influence the outcome of chemotherapy. Preliminary results with JM8 suggest that it is an active single agent in the treatment of seminoma.

Approximately 70% of patients with testicular seminoma present without clinical evidence of metastases (Stage I) and in the majority of the remainder metastases appear confined to infradiaphragmatic nodes (Stage II) (Peckham, 1981). The early stage presentation together with the radiosensitivity of seminoma has resulted in excellent survival figures (Maier *et al.*, 1968; Smithers *et al.*, 1971; Castro & Gonzales, 1971; Doornbos *et al.*, 1975; Kademian *et al.*, 1976; Van der werf Messing, 1976; Calman *et al.*, 1979; Thomas *et al.*, 1982). However, despite these good overall results, subgroups can be identified where the results of radiotherapy are less satisfactory. These include patients with bulky Stage II disease and Stage III and IV presentations (Ball *et al.*, 1982).

Experience in recent years has shown that seminomas are responsive to the chemotherapy used to treat non-seminomatous germ-cell tumours, suggesting that chemotherapy could be considered as an alternative to or in conjunction with radiotherapy in selected patients (Einhorn & Williams, 1980; Ball *et al.*, 1982; Morse *et al.*, 1983; Simon *et al.*, 1983; Van Oosterom *et al.*, 1984).

### Patients and methods

### *Patients*

Between October 1978 and November 1983, 44 patients aged from 23 to 56 years were treated with chemotherapy containing cis-platinum (39 patients) or the platinum analogue cis-diammine-1, 1-cyclobutane dicarboxylate platinum II (CBDCA or JM8) as a single agent (5 patients). No patient had received prior chemotherapy, but 13 had been irradiated and subsequently relapsed. Of the total group, 36 patients had primary intra-scrotal testicular seminomas and 1 patient had a massive seminoma in an undescended abdominal testis. Four patients (all male) had primary mediastinal seminomas, in two with lung infiltration and in one with involvement of a vertebra and extradural cord compression. Three patients had no detectable primary tumour but on the basis of the pattern of spread were presumed to have occult seminomas in the testis.

The observation time from start of chemotherapy for the series is 12 to 73 months, median 36 months.

### *Staging*

This included chest X-ray, lymphography, CT scans of chest and abdomen, i.v. urography and in selected patients Gallium 67 scanning. Serum concentrations of alphafoetoprotein (AFP) and human chorionic gonadotrophin (HCG) were measured initially in all patients and employed as a monitor of disease progress thereafter.

The Royal Marsden staging classification (Peckham *et al.*, 1979) was employed:

Stage I:
  No clinical evidence of metastases.
Stage II:
  Infradiaphragmatic lymph node involvement
    A – maximum diameter $<2$ cm
    B – maximum diameter 2–5 cm
    C – maximum diameter $>5$ cm.

Correspondence: M.J. Peckham.
Received 28 November 1984; and in revised form 5 March 1985.

© The Macmillan Press Ltd., 1985

Stage III:
Supra and infradiaphragmatic node involvement.
A, B and C as for II.

Stage IV:
Extranodal metastases A, B and C as for II.
Lung sub-staging –
$L_1 \leqslant 3$ metastases
$L_2 > 3$ metastases all $< 2$ cm diameter
$L_3 > 3$ metastases one or more $> 2$ cm diameter.
H+ liver involvement other sites, e.g. bone denoted.

*Criteria for entry to study*

All patients had histologically proven pure seminoma. Tissue stains for AFP were negative and serum AFP concentrations within normal limits. During the period of study there were 8 patients with histologically pure seminoma who had either a raised serum alphafoetoprotein titre (7 patients) or high level of HCG (1 patient; HCG 23,000 iu $l^{-1}$). These were excluded. Patients who had had prior chemotherapy were excluded. Patients with Stages IIC, III and IV were included in the study. All but 4 Stage IIA and IIB patients were excluded since these were treated with infradiaphragmatic irradiation. Of the 4 IIA/IIB patients in the present study 2 had had prior irradiation and 1 was not irradiated because an accident in childhood has resulted in extensive scarring to the abdominal wall and groin. The fourth had a 5 cm diameter mass and received chemotherapy.

*Treatment*

Chemotherapy in the initial period of the study consisted of cis-platinum, vinblastine and bleomycin (PVB) (Einhorn & Donohue, 1977). Subsequently, bleomycin, etoposide and cis-platinum (BEP) (Peckham *et al.*, 1983) were employed. Etoposide and cis-platinum were employed in 1 patient. JM8 was employed in 5 patients as a single agent in a dose of 400 mg $m^{-2}$ given as a 30 min i.v. infusion every 3–4 weeks (Calvert *et al.*, 1982). Four patients had 4 doses and one had 6. A sixth patient received 4 injections of JM8 for a mediastinal seminoma with lung infiltration followed by 2 cycles of BEP, since despite an excellent response, residual thickening was present. Of the 44 patients, 31 (70%) received 4 cycles of chemotherapy, two had 2 cycles, three 5 cycles, seven 6 cycles and one 8 cycles. The number of patients receiving each type of chemotherapy is shown in Table I.

Between 1978 and 1982 all but 7 patients had involved site irradiation after chemotherapy. The 7 who did not, included one patient who had undergone an extensive resection of an intra-abdominal testis involving bladder and bowel, a patient with Down's Syndrome treated with JM8, a patient with lung infiltration and a primary mediastinal seminoma, two patients with IIC disease and one patient with IIIC disease.

**Table I** Advanced seminoma: Outcome of treatment by type of chemotherapy
(The Royal Marsden Hospital, 1978–1983)

| *Chemotherapy* | *No. of patients* | *Relapses* |
|---|---|---|
| Bleomycin, etoposide, cis-platinum | 25 | 1 |
| Etoposide, cis-platinum | 1 | 0 |
| Cis-platinum, vinblastine, bleomycin | 8 | 1 |
| Carboplatin | 5 | 1 |
| Combination of above | 5 | 2 |
| Total | 44 | 5 |

*Assessment of response and follow-up*

One month after completion of chemotherapy patients were reassessed with chest and abdominal X-rays, CT scans of chest and abdomen, measurement of serum AFP and HCG concentrations and, where indicated, i.v. urography and repeat Gallium 67 scans. A complete response was indicated by the total absence of disease as judged clinically, radiologically and biochemically. If residual masses were identified these were monitored carefully at subsequent follow-up visits. AFP and HCG serum levels were measured at each visit in all patients.

### Results

Of the 44 patients, 40 (90%) are alive and 4 have died of germ-cell malignancy, 2 with non-seminomatous metastases. Of those who are alive, 39 have remained continuously disease free for 12–73 months (median 36 months) since treatment and one is disease free 17 months after relapse following initial chemotherapy.

*Results by type of chemotherapy*

As shown in Table I the results obtained with the different approaches used are comparable.

*Influence of prior radiotherapy*

Of 13 patients who had been previously irradiated 3 (23%) have died compared with 1/31 (3%) previously untreated patients (Table II). The results

**Table II** Advanced seminoma: Outcome of chemotherapy in patients relapsing after radiotherapy and previously untreated patients
(The Royal Marsden Hospital, 1978–1983)

| *Prior radiotherapy* | *No. of patients* | *No. relapsing* | *Dead* |
|---|---|---|---|
| Yes | 13 | 3 (23%) | 3 (23%)[a] |
| No | 31 | 2 (6%) | 1 (3%) |
| Total | 44 | 5 (11%) | 4 (9%) |

[a]$P<0.03$.

indicate a better survival for chemotherapy when no prior radiotherapy was received by the patient (log rank test $\chi^2=4.60$, $P=0.03$).

*Influence of tumour bulk*

Table III shows the outcome of treatment in relation to clinical stage in previously untreated patients. Although 2 relapses occurred in patients with bulky abdominal disease the overall results in patients with extensive disease including bone and liver were good, indicating that tumour volume exerts less effect on treatment outcome with the type of chemotherapy employed than is the case for non-seminomatous tumours. Of the 5 patients receiving JM8 as a single agent, 4 had bulky disease (IIC or IIIC) of whom one failed and one had small volume disease ($IVOL_1$) and is in complete remission.

**Table III** Advanced seminoma: Outcome of chemotherapy by stage in previously untreated patients
(The Royal Marsden Hospital, 1978–1983)

| *Stage/site presentation* | *No. of patients* | *Continuously disease-free*[a] | *Deaths* |
|---|---|---|---|
| Primary mediastinal | 1 | 1 | |
| Primary mediastinal +lung | 2 | 2 | |
| IIA/IIB | 4 | 4 | |
| IIC | 17 | 15 | 1 |
| IIIC | 4 | 4 | |
| IV[a] | 3 | 3 | |
| Total | 31 | 29 (93%) | 1 (3%) |

[a]One patient who relapsed and is disease-free after salvage treatment is not included.

[b]Primary mediastinal seminoma with bone and extradural cord compression; disease-free 19 months. Testicular primary with bone and liver involvement; disease-free 65 months. Intra-abdominal testis with local extension into bowel and bladder; disease-free 29 months.

*Influence of post-chemotherapy radiotherapy (Table IV)*

Of 15 previously untreated patients receiving involved site radiotherapy after chemotherapy, one relapsed. Similarly there was only one relapse in 16 patients treated with chemotherapy alone. Although follow-up times are shorter in the latter group (median 19 months compared with 43 months), it is unlikely that any significant differences will emerge between these two approaches.

**Table IV** Advanced seminoma: Results of chemotherapy ±adjuvant radiotherapy
(The Royal Marsden Hospital, 1978–1983)

| *Elective involved site irradiation after chemotherapy* | *No. of patients* | *No. relapsing* | *Observation time (months)* Range | Median |
|---|---|---|---|---|
| Yes | 15 | 1 | 21–73 | 43 |
| No | 16 | 1 | 12–65 | 19 |

*Assessment of complete response*

The results of reassessment one month following completion of chemotherapy showed that only 14/44 (32%) had achieved complete clearance of tumour. As shown in Table V when complete response rates were related to bulk of disease most complete responses were seen in patients with small volume disease and the rate in bulky disease patients was only 12%.

*Surgery after chemotherapy*

Four patients were explored after chemotherapy for residual masses. All 4 had fibrotic masses with no evidence of malignancy. One of the 4 patients subsequently relapsed with an elevated serum AFP level and at autopsy had undifferentiated malignant teratoma metastases.

*Patterns of and time to relapse*

Details on 5 relapsing patients are shown in Table VI. In all cases relapse appeared in initially involved sites and in one patient relapse occurred after 2 years. In 2 patients relapse was associated with raised serum AFP levels.

*Pre-treatment serum HCG levels*

As shown in Table VII, only 11% of patients had completely normal serum HCG concentrations, although in 52% of patients, the levels were low. The highest level seen was $661\,iu\,l^{-1}$. Pre-

**Table V** Advanced seminoma: Disparity between conventional response assessment and subsequent outcome
(The Royal Marsden Hospital, 1978–1983)

| *Volume of tumour at presentation* | *No. of patients* | *Complete disappearance of tumour by one month post chemotherapy* | *Subsequent relapses* |
|---|---|---|---|
| Non-bulky[a] | 11 | 10 (91%) | 1 (9%) |
| Bulky | 33 | 4 (12%) | 4 (12%) |
| Total | 44 | 14 (32%) | 5 (11%) |

[a]Bulky defined as abdominal status C, lung status $L_3$ or mediastinal mass > 5 cm diameter.

**Table VI** Advanced seminoma: Details of relapse after chemotherapy
(The Royal Marsden Hospital, 1978–1983)

| *Patient* | *Prior irradiation* | *Stage* | *Serum HCG level before chemo.* ($iu\,l^{-1}$) | *Chemotherapy* | *Months to relapse* | *Site(s)* | *Markers at relapse* *AFP* ($ng\,ml^{-1}$) | *HCG* ($iu\,l^{-1}$) |
|---|---|---|---|---|---|---|---|---|
| 1 | + | IIC | 8 | PVB | 15 | ABDO | 100 | <1 |
| 2 | + | IIB | 2 | BEP | 5 | ABDO | 63 | <1 |
| 3 | | IIC | 4 | BEP | 26 | ABDO | <5 | 3 |
| 4 | | IV bone/ liver | 2 | PVB/BEP | 3 | Bone | <5 | 3 |
| 5 | | IIC | 9 | JM8 | 1 | ABDO | <5 | 6 |

**Table VII** Advanced seminoma: Serum HCG levels prior to chemotherapy
(The Royal Marsden Hospital, 1978–1983)

| *Serum HCG level* ($iu\,l^{-1}$) | *No. of patients* | % |
|---|---|---|
| <1 | 5 | 11 |
| 1–9 | 23 | 52 |
| 10–19 | 3[a] | |
| 20–49 | 6[a] | |
| 50–99 | 3[a] | 37 |
| 100–199 | 2[a] | |
| 200–300 | 2[a] | |

[a]Relapsing patients.

chemotherapy HCG levels were low in all 5 relapsing patients (Table VI) suggesting that this is not a significant prognostic factor at least when combination chemotherapy is employed.

*Toxicity*

There were no deaths attributable to the effects of chemotherapy. The toxicity of PVB, BEP and EP has been described elsewhere (Einhorn and Donohue, 1977; Peckham *et al.*, 1983; Peckham & Horwich, 1985). Of the 5 patients treated with JM8, 2 experienced nausea and vomiting with each injection, 2 experienced some nausea but did not vomit and one was asymptomatic for two injections and experienced nausea and vomiting with two. Hair loss did not occur and there was no impairment of renal function or peripheral neuropathy. Nadir blood counts were as follows: White count, $2\text{–}3.9 \times 10^3\,ml^{-1}$; platelets, $37\text{–}62 \times 10^3\,ml^{-1}$ and haemoglobin, $9.3\text{–}13.4\,G\,l^{-1}$. The gaps between injections of JM8 were as follows: <21 days, 6 gaps; 22–28 days, 7 gaps; 29–35 days, 2 gaps with one interval of 36 days and one of 39 days.

### Discussion

The present results together with those reported from other centres show that seminoma is highly sensitive to platinum-containing chemotherapy (Table VIII). More limited data indicate that platinum analogues are active as single-agents. As shown in Table IX Samuels *et al.* (1983) have

**Table VIII** Advanced seminoma: Results of treatment with cis-platinum containing combination chemotherapy

| *Authors* | *Drugs* | *No. of patients* | *No. achieving complete remission (CR) (%)* | *Currently disease free (%)* | *No. of relapses in CR patients (%)* | *Time to relapse (months)* |
|---|---|---|---|---|---|---|
| Van Oosterom *et al.* (1984)[a] | Cis-platinum + vinblastine bleomycin ± adriamycin | 73 | 51 (70) | NS | NS | NS |
| Oliver (1984) | Cis-platinum + vinblastine bleomycin | 12 | NS | 10 (83) | NS | NS |
| Morse *et al.* (1983) | Cis-platinum + vinblastine actinomycin-D cyclophosphamide bleomycin | 22 | 19 (86)[b] | 18 (82) | 1/19 (5) | NS |
| Simon *et al.* (1983) | Cis-platinum + vinblastine antinomycin-D, cyclophosphamide bleomycin | 10 | 10 (100) | 10 (100) | 3/10 (30) | 6, 7, 8 |
| Peckham *et al.* (present series) | Cis-platinum + bleomycin etoposide and/or vinblastine | 39 | 13 (33) (see text) | 36 (92) | 0/13 | |

[a]Collected data from Indiana, Netherlands and Madrid.
[b]Includes surgical and clinical assessment, using clinical criteria only 9/22 (40%) achieved CR.

**Table IX** Advanced seminoma: Outcome of treatment with platinum analogues as single agents

| *Authors* | *Chemotherapy* | *No. of patients* | *No. disease-free* |
|---|---|---|---|
| Samuels *et al.* (1983) | Cis-platinum | 32 | 30 (22 > 2 years) |
| Oliver (1984) | Cis-platinum | 14 | 10 (median follow-up 14 months) |
| Peckham *et al.*[a] (present series) | JM8[b] | 7 | 6 (6–32 months) (median 15 months) |

[a]Includes two further patients observed for < 1 year.
[b]One patient received radiotherapy after JM8.

reported 32 patients treated with cis-platinum ± cyclophosphamide of whom 30 are in continuing complete remission, 27 for more than 18 months. In their study cis-platinum was given weekly but it is not clear how many patients also had cyclophosphamide. Oliver (1984) has reported on 14 patients treated with cis-platinum alone of whom 10 are disease free. Of 10 untreated patients 9 are disease free. Preliminary data from the present study employing JM8 as a single agent is encouraging with 4/5 patients disease free at 12, 16, 28 and 32 months. A further patient disease free at 14 months achieved an excellent response with 4 doses of JM8 and then received 2 cycles of BEP as consolidation because of residual mediastinal widening. Obviously further information is required before the role of carboplatin in the management of seminoma can be assessed.

A potential disadvantage of employing single agent chemotherapy in seminoma is the possibility that unrecognized non-seminomatous tumour components may be present but are not subject to control resulting in drug resistant tumour at relapse. In the present series 2/39 patients treated with combination chemotherapy had raised serum AFP levels at relapse. None of the patients treated with JM8 as a single agent have relapsed as a non-seminoma. It is not stated whether the two deaths in the series reported by Samuels and his colleagues were treatment-related or due to seminoma or non-seminoma, however, 30/32 are disease free which suggests that the risk of unrecognized and uncontrolled non-seminomatous components is unlikely to pose a major problem. The present data show that moderate elevation of serum HCG levels is common in metastatic seminoma and appears not to influence the results of combination chemotherapy. Whether this will remain true for single agent platinum chemotherapy remains to be demonstrated.

So far as the use of combination chemotherapy is concerned, the apparent effectiveness of platinum analogues as single agents raises doubts about the contribution of other drugs included in the regimens summarized in Table VIII. Samuels *et al.* (1983) have reported on 18 patients treated with bleomycin, cyclophosphamide, vincristine, methotrexate and 5 fluorouracil of whom 10 achieved CR but five subsequently failed. All 7 patients treated with vinblastine and bleomycin failed to achieve CR and died. Since the latter combination resulted in CR rates of $\sim 50\%$ in patients with non-seminomatous germ-cell tumours, it appears that the spectrum of drugs active in seminoma and non-seminoma may differ substantially. A major difference between the present series and those previously reported is the complete response (CR) rate of 32% which is considerably lower than the figure obtained at other centres. This difference is likely to reflect differences in the time at which response was assessed as well as the methods of assessment. It is clear that other centres have also observed residual masses after chemotherapy. Thus in the memorial series of 22 patients, only 9 (40%) achieved complete disappearance of masses and 10 were submitted to surgery for residual abnormalities (Morse *et al.*, 1983). All 10 patients were histologically negative. Of the 10 patients reported by Simon *et al.* (1983) seven were explored after chemotherapy and all were histologically negative.

It is clear that residual masses are common after completion of chemotherapy for advanced seminoma, particularly if disease is bulky initially. These masses resolve slowly over months, and in some instances, years. Surgical exploration is complicated by the densely adherent fibrotic nature of the residuum and generally proves negative. In addition to the 17 patients undergoing laparotomy as described above four patients in the present series were explored and all were negative. Surgery is therefore not advocated, although careful monitoring of residual masses is essential. In this context preliminary data indicate that placental alkaline phosphatase is likely to prove a useful marker in seminoma (Lange *et al.*, 1982).

Recurrences can occur in patients achieving complete remission and those who are histologically negative at surgery. As shown in Table VIII 1/19 CR patients in the series of Morse *et al.* (1983) relapsed as did 3/10 patients reported by Simon *et al.* (1983). In the latter group 3/7 patients, who were histologically negative at surgery relapsed as did 1/4 in the present series. None of 10 surgically negative patients reported by Morse relapsed.

Although the role of adjuvant radiotherapy after chemotherapy has not been formally investigated in a randomized trial, limited data summarized in Table IV suggest that it is unnecessary. Gradual resolution of residual masses occurs and as noted above the histology of resected tissue has generally proved negative.

To date there is scanty information on time to relapse after combination chemotherapy for advanced seminoma. Relapses in the present series occurred at 1, 3, 5, 15 and 26 months after chemotherapy and in the series of Simon *et al.* (1983) at 6, 7 and 8 months. Hence 6/8 relapses have been within the first year of chemotherapy.

In conclusion, seminoma is a highly chemoresponsive tumour in which the influence of tumour volume on the outcome of chemotherapy appears to be less obvious than is the case for non-seminomas although residual masses are commonly present one month after completion of chemotherapy. The results of surgery show that such masses are almost invariably negative histologically and often densely fibrotic. Spontaneous resolution of residual masses occurs, often over a period of months or years. There is no evidence that post-chemotherapy radiotherapy is contributory.

The authors are grateful to Dr A.H. Calvert for supplying JM8, to Julie Butcher for preparing the manuscript and Gillian Jay and Judy Nicholls for their help in data collection.

### References

BALL, D., BARRETT, A. & PECKHAM, M.J. (1982). The management of metastatic seminoma testis. *Cancer*, **50**, 2289.

CALMAN, F.M.B., PECKHAM, M.J. & HENDRY, W.F. (1979). The pattern of spread and treatment of metastases in testicular seminoma. *Br. J. Urol.*, **51**, 154.

CALVERT, A.H., HARLAND, S.J., NEWELL, D.R. & 9 others. (1982). Early clinical studies with cis-diammine-1, 1-cyclobutane dicarboxylate platinum II. *Cancer Chemother. Pharmacol.*, **9**, 140.

CASTRO, J.R. & GONZALES, M. (1971). Results in treatment of pure seminoma of the testis. *Am. J. Roentgenol*, **III**, 355.

DOORNBOS, J.F., HUSSEY, D.H. & JOHNSON, D.E. (1975). Radiotherapy for pure seminoma of the testis. *Radiology*, **116**, 401.

EINHORN, L.H. & DONOHUE, J.P. (1977). Cis-diammine-dichloroplatinum, vinblastine and bleomycin combination chemotherapy in disseminated testicular cancer. *Ann. Intern. Med.*, **87**, 293.

EINHORN, L.H. & WILLIAMS, S.D. (1980). Chemotherapy of disseminated seminoma. *Cancer Clin. Trials*, **3**, 307.

KADEMIAN, M.T., BOSCH, A. & CALDWELL, W.L. (1976). Seminoma: Results of treatment with megavoltage irradiation. *Int. J. Radiat. Oncol. Biol. Phys.*, **1**, 1075.

LANGE, P.H., MILLAN, J.L., STIGBRAND, T., VESSELLA, R.L., RUOSLAHTI, E. & FISHMAN, W.H. (1982). Placental alkaline phosphatase as a tumour marker for seminoma. *Cancer Res.*, **42**, 3244.

MAIER, J.G., SULAK, M.H. & MITTEMEYER, B.T. (1968). Seminoma of the testis: Analysis of treatment success and failure, *Am. J. Roentgenol.*, **102**, 596.

MORSE, M., HERR, H., SOGANI, P., BOSL, G. & WHITMORE, W.F. (1983). Surgical exploration of metastatic seminoma following VAB VI chemotherapy., *Proc., Am. Soc. Clin. Oncol.*, **2**, 143 (Abstract).

OLIVER, R.T.D. (1984). Surveillance for Stage I seminoma and single agent cis-platinum for metastatic seminoma. *Proc., Am. Soc. Clin. Oncol.*, **3**, 162 (Abstract).

PECKHAM, M.J. (1981). In *The Management of Testicular Tumours*. (Ed. Peckham) Edward Arnold Ltd., London, p. 134.

PECKHAM, M.J., BARRETT, A., LIEW, K.H. & 5 others. (1983). The treatment of metastatic germ-cell testicular tumours with bleomycin, etoposide and cis-platin (BEP). *Br. J. Cancer*, **47**, 613.

PECKHAM, M.J., BARRETT, A., McELWAIN, T.J. & HENDRY, W.F. (1979). Combined management of malignant teratoma of the testis. *Lancet*, **ii**, 257.

PECKHAM, M.J., HORWICH, A., BLACKMORE, C. & HENDRY, W.F. (1985). Etoposide and cis-platin with or without bleomycin as first line chemotherapy for patients with small volume metastases of testicular non-seminoma. *Cancer Treatment Rep.* (in press).

SAMUELS, M.L. & LOGOTHETIS, C.J. (1983). Follow up study of sequential weekly pulse dose cis-platinum for far advanced seminoma. *Proc., Am. Soc. Clin. Oncol.*, **2**, 137 (Abstract).

SIMON, S.D., SROUGI, M. & GOES, G.M. (1983). Treatment of advanced seminoma with vinblastine, actinomycin-D, cyclophosphamide, bleomycin and cis-platinum. *Proc., Am. Soc. Clin. Oncol.*, **2**, 132.

SMITHERS, D.W., WALLACE, E.N.K. & WALLACE, D.M. (1971). Radiotherapy for patients with tumour of the testicle. *Br. J. Urol.*, **43**, 83.

THOMAS, G.M., RIDER, W.D., DEMBO, A.J. & 5 others. (1982). Seminoma of the testis: Results of treatment and pattern of failure after radiation therapy. *Int. J. Radiat. Oncol. Biol. Phys.*, **8**, 165.

VAN DER WERF MESSING, B. (1976). Radiotherapeutic treatment of testicular tumours. *Int. J. Radiat. Oncol. Biol. Phys.*, **I**, 235.

VAN OOSTEROM, A.T., WILLIAMS, S.D., CORTES FUNES, H., TEN BOKKEL HUININK, W.W. & VENDRIK, C.P.J. (1984). Treatment of seminomas with chemotherapy. In *Progress and Controversies in Oncological Urology*. (Ed. Kurth) Alan R. Liss Inc., 103.